# Triboelectric Nanogenerator Enabled Smart Shoes for Wearable Electricity Generation

**DOI:** 10.34133/2020/7158953

**Published:** 2020-11-09

**Authors:** Yongjiu Zou, Alberto Libanori, Jing Xu, Ardo Nashalian, Jun Chen

**Affiliations:** Department of Bioengineering, University of California, Los Angeles, Los Angeles, CA 90095, USA

## Abstract

The parallel evolution of wearable electronics, artificial intelligence, and fifth-generation wireless technology has created a technological paradigm with the potential to change our lives profoundly. Despite this, addressing limitations linked to continuous, sustainable, and pervasive powering of wearable electronics remains a bottleneck to overcome in order to maximize the exponential benefit that these technologies can bring once synergized. A recent groundbreaking discovery has demonstrated that by using the coupling effect of contact electrification and electrostatic induction, triboelectric nanogenerators (TENGs) can efficiently convert irregular and low-frequency passive biomechanical energy from body movements into electrical energy, providing an infinite and sustainable power source for wearable electronics. A number of human motions have been exploited to properly and efficiently harness this energy potential, including human ambulation. Shoes are an indispensable component of daily wearing and can be leveraged as an excellent platform to exploit such kinetic energy. In this article, the latest representative achievements of TENG-based smart electricity-generating shoes are comprehensively reviewed. We summarize ways in which not only can biomechanical energy be scavenged via ambulatory motion, but also biomonitoring of health parameters via tracking of rhythm and strength of pace can be implemented to aid in theranostic fields. This work provides a systematical review of the rational structural design, practical applications, scenario analysis, and performance evaluation of TENG-based smart shoes for wearable electricity generation. In addition, the perspective for future development of smart electricity-generation shoes as a sustainable and pervasive energy solution towards the upcoming era of the Internet of Things is discussed.

## 1. Introduction

It is no secret that the Internet of Things is changing how business is conducted and how life is lived in fundamental and meaningful ways. Distributed electronics are the key components for enabling any Internet of Things applications via means of a wide range of engineered solutions including sensing [1–6], therapy [7–9], and environmental monitoring [10–12]. However, ensuring constant power sources to feed these distributed electronics, including stand-alone devices, is an essential prerequisite currently beyond the capability of traditional centralized power supply systems [13–16]. Traditional batteries have limited lifetime, rigid structures, inconvenient heaviness, and can lead to potential environmental pollution, thus alternatives are required [17–22]. The human body inherently generates a large amount of biomechanical energy via daily activities such as walking and running, providing a rich source of renewable energy [23–29]. For example, ambulatory footfall generates as much energy as 20 W. [30–33]. If this energy were to be harnessed to power wearable electronics, it could ensure life-long operations in a sustainable and independent way, eliciting much interest in establishing a human footfall energy harvesting system. Even though converting human footfall energy could provide a superior solution to obtain sustainable energy for on-body electronics powering, the increase in population aging opens up unmet clinical needs which ambulatory monitoring can help address, including gait [34–37]. Effective gait monitoring can be used in many health-related scenarios, such as the detection of sudden falls [38–40], leg rehabilitation assessments [41–44], and detection and progression of Parkinson's disease [45–48]. Current ambulatory monitoring methods such as mobile phones and sports bracelets can be used to track the number of steps walked, but fail to provide insight into clinical conditions. Devices which can specifically monitor gait are expensive and cannot be easily used at anytime and anywhere, because of the limited time and space. To overcome this challenge, a number of progressive smart shoes have been shown to effectively convert human footfall into electric signals as a convenient and cost-effective approach for both energy harvesting and active sensing purposes [49–54]. A variety of smart shoes are developed via engineering different working mechanism with which they convert biomechanical energy into electricity, including piezoelectric approaches [55–58], electromagnetic approaches [59, 60], and many others [61–63].

**Figure 1:**
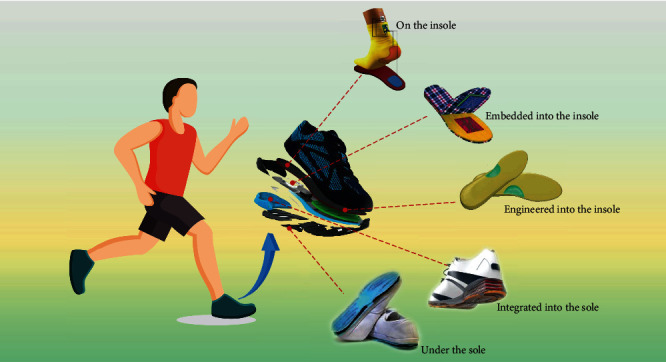
Diverse applications of TENG enabled smart shoes for mechanical-to-electrical energy conversion. On the insole. Reproduced with permission from [129]. Copyright Elsevier, 2017. Embedded into the insole. Reproduced with permission from [130]. Copyright Elsevier, 2017. Engineered into the insole. Reproduced with permission from [131]. Copyright Royal Society of Chemistry, 2019. Integrated into the sole. Reproduced with permission from [132]. Copyright Springer Nature, 2015. Under the sole. Reproduced with permission from [133]. Copyright Springer Nature, 2016.

In 2012, the triboelectric nanogenerators emerged as a compelling means to generate electricity based on the conjunction of the triboelectrification and electrostatic induction [64–66]. TENGs' merits have been well documented, and comprise high efficiency, low cost, light weight, simple structure, biocompatibility, and wide-range of materials choices [67–77]. TENGs were successfully applied to harvest mechanical energy from sound [78–82], wind [83–90], water waves [91–96], and vibration [97–101]. Owing to their unique working mechanism and material usage, TENGs can be flexible [102–107], stretchable [108–115], humidity-proof [116, 117], self-healing [118–120], shape-adaptive [121–123], and even washable [124–128]. In view of these advantages, this promising technology has been cleverly applied to different parts of shoes, creating TENG-based smart footwear for biomechanical energy harvesting, as shown in Figure 1.

In this review, we first discuss the selection of triboelectric materials and the working principle of TENG for smart electricity-generating shoes. Subsequently, emphasis is placed on the recent progress and practical applications of TENG-based smart shoes for electricity generation, classified according to the operating locations in the shoes. Finally, perspectives and challenges for the future development of wearable TENGs are discussed. We hope that this review will significantly promote the development of wearable TENG-based smart shoes and shed light on providing a pervasive and sustainable energy solution to the wearable electronic systems in the era of the Internet of Things.

## 2. Working Mechanism

**Figure 2:**
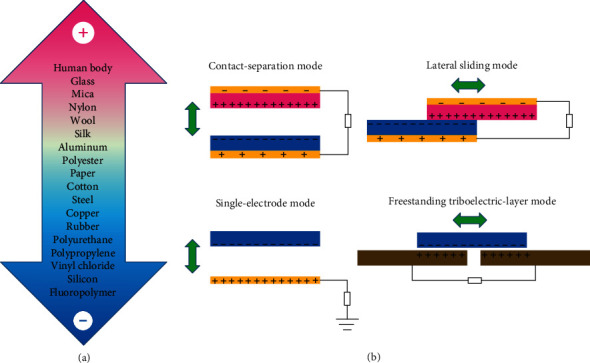
Working principle of TENG. (a) Triboelectric series depending on their electron affinity. (b) Four fundamental operation modes of TENG.

The triboelectric effect is often considered as negative or even hazardous in daily life, because it is not only an irritating event, but it allows for dust to collect everywhere and, in extreme cases, can lead to dangerous events, including fires. Despite this, TENGs can take advantage of usually wasted and ubiquitously present ambient mechanical energy and convert it into electric energy using the coupling of contact electrification and electrostatic induction processes [66, 134–136]. The triboelectric effect results from the cyclic contact and separation of two different materials with different electron affinity, which is defined as the “triboelectric series,” as shown in Figure 2(a). The lower the material's position in the series, the better its ability to obtain electrons and get negatively charged, and the further apart the two materials in the triboelectric series, the more transfer charges are generated during the physical contact [137–140]. Consquently, the “triboelectric series” can be employed as the guideline in the selection of optimized triboelectric material pairs for high output performance, prior to designing and fabricating novel TENGs. Tribo-materials selected for the fabrication of TENG are ubiquitously present in our daily lives [141–146], among them, metal and nylon are commonly positively charged, while silicone rubber and polytetrafluoroethylene (PTFE) are typically chosen as negative materials for gaining negative charges [147–150].

When two triboelectric materials with different electron affinities come into physical contact, tribo-charges are separated and transferred from one material to the other. The surface of the material with higher electron affinity becomes negatively charged, while the other surface becomes positively charged with an equal amount. When the two materials separate, the tribo-charges in the interfacial regions too are separated, inducing an electrical potential difference between electrodes, and driving free electrons to flow back and forth in the external circuit to maintain the electrostatic equilibrium. The fundamental working modes of TENG can be divided into the following four categories: vertical contact-separation mode [151], lateral sliding mode [152], single-electrode mode [153], and freestanding triboelectric-layer mode [154], depending on the structure and relative motion of the materials used (Figure 2(b)).

## 3. Smart Shoes for Biomechanical Energy Harvesting

Recently, tremendous efforts have been devoted to developing wearable TENGs to be worn as on body bioelectronics or integrated into clothes and accessories. Amongst those, TENG-based smart shoes attracted public attention on account of their ability to easily and conveniently convert biomechanical energy generated during regular walking, into electric energy, so that it may be used for third party devices. Since TENGs could be miniaturized with thin and lightweight materials, they can easily be placed just below the foot as part of smart shoes, and harness passive biomechanical ambulatory motions to generate electricity. Insoles are one of the most popular components of shoes which have been integrated with TENG for wearable electricity generation. Studies have demonstrated that one walking step can produce 1 to 5 joules of energy [155]. If this pervasive energy source is harnessed effectively, it can hold the potential to capture a large amount of passively available energy, with obvious sustainability benefits. To elucidate the working principle of TENG-based smart shoes, a detailed explanation of how TENG-based smart shoes generate electricity is provided below, with the TENG position varying from shoe top to shoe bottom.

### 3.1. TENGs Mounted on the Insoles

**Figure 3:**
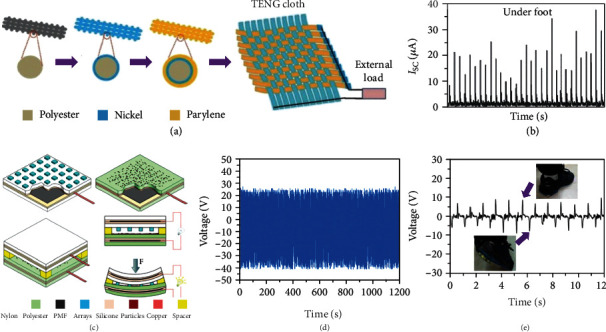
Textile-based TENGs mounted on the insoles. (a) Schematic illustration of the fabrication of TENG-cloth. (b) The short current when the TENG-cloth was worn under the foot. Reproduced with permission from [160]. Copyright Wiley-VCH, 2015. (c) Schematic of the structure and working mechanism of the textile-based TENGs. (d) Output voltage of the textile-based TENG tested for 1200 s and over 7,200 cycles at the frequency of 6 Hz. (e) Output voltage generated by the textile-based TENG mounted on the insole. The two insets exhibit the shoe to extract biomechanical energy from human footfall. Reproduced with permission from [158]. Copyright Springer Nature, 2019.

TENGs mounted directly on the insole are very simple and convenient as they require no intermediary processing. The TENG is in direct contact with the feet (or socks), so the triboelectric materials used needs to be of a soft, skin-friendly, waterproof, dirt-resistant, and bacteria-resistant nature[129, 156–160]. Pu et al. proposed a wearable textile TENG-cloth mounted directly on the insole yielding a solution with flexibility, comfortability, air-breathability, and water-washability [160].  Figure 3(a) shows the schematic illustration of the fabrication of the said TENG-cloth. The soft polyester fabric was chosen as the base substrate and coated with the Ni film and parylene film, forming a conductive Ni-cloth, and insulating parylene-cloth layers. The belt-type Ni-cloth and parylene-cloth were woven into a 5 cm × 5 cm TENG-cloth, of which every basic unit was composed of belt-type Ni-cloth, using Ni film as both the triboelectric layer and electrode, and belt-type parylene-cloth using Ni film as electrode, and parylene film as the other triboelectric layer. Owing to the ingenuity of the structural design, this TENG-cloth can work in a variety of modes, including vertical contact-separation mode and horizontal sliding mode. In the contact-separation mode, the TENG-cloth and skin (or other ordinary clothes) can be used as a pair of triboelectric layers, as can two identical TENG-cloths. As to the sliding mode, two identical TENG-cloths produce frictional motion in contact with each other, which causes the transfer of free electrons betwen the materials, due to different electron affinity of the materials. As shown in Figure 3(b), when the TENG-cloth is worn under the foot, electricity can be generated by walking, and used to light up to 37 LEDs. The TENG-cloth was also used in scavenging biomechanical energy to charge a Li-Ion Battery (LIB) belt, powering a heartbeat meter. The invention of wearable TENG-cloth can inspire us to come up with a number of innovative ideas for the applications as wearable electronics in the near future. Chang et al. developed yet another textile-based TENG, integrated with plastic metal electrodes, and employing the contact-separation mode [158]. The schematic of the structure and working mechanism of the textile-based TENGs is shown in Figure 3(c). The triboelectric layer pair was made up of nylon textile embroidered with convex arrays in circular or square patterns on the surface, and polyester textile coated with polyvinylidene fluoride (PVDF) based nanofibers and particles, to obtain nanostructures on the surface, which all increase the contact extents on the triboelectric surface, enhancing output performance. The PMFs made from Ga-In liquid alloy-added glaze powders-a type of plastic metal using a coating scraper- were selected as the electrodes of the triboelectric layers. To prevent the liquid electrode from leaking, the backside of nylon and polyester textiles was coated with silicone films, and the copper wires were connected to the PMFs as the leads of electrodes. Finally, four elastic sponges fixed at four corners between two triboelectric layers, were used to restore TENG's shape after compression. In this example, the working mechanism depended on cyclic contact and separation between two triboelectric layers.  Figure 3(d) illustrates how the textile-based TENG had been tested for 1200 s and over 7200 cycles at a frequency of 6 Hz, wihtout showing any considerable decrease in the output voltage, proving this textile-based TENG could be used as a very stable and durable option. The textile-based TENG was mounted onto the insole as shown in Figure 3(e), and the maximum output voltage generated reached circa 10 V while walking.

**Figure 4:**
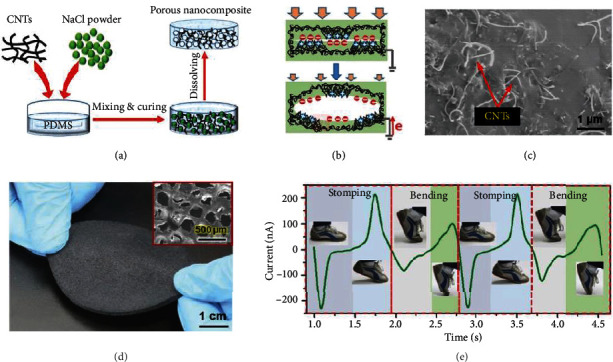
Porous TENG mounted on the insoles. (a) Diagram of the synthesis process. (b) The process of generating electricity from a single cavity. (c) SEM magnified view of the inner surface, with exposed CNTs labeled. (d) Photograph of an as-prepared round PNC (5 cm in diameter and 0.5 cm thick). The SEM inset shows the surface morphology. (e) Cycled *I*_SC_ and corresponding status of a foot under repeated normal walking. Reproduced with permission from [159]. Copyright Wiley-VCH, 2016.

On the multilayer structure front, garment-based TENGs were developed using the contact-separation mode which included fabrication modifications to improve output performance [156]. Also, a laser-induced graphene-based TENG employing the single-electrode mode was developed, yielding open-circuit output voltage of above 3.5 kV [157]. Amongst the most innovative work in this field, we highlight a stretchable porous nanocomposite (PNC) based on a hybrid of a polydimethylsiloxane(PDMS) matrix and a multiwalled carbon nanotubes (CNTs) network used to harvest energy from biomechanical motions [159].  Figure 4(a) illustrates the fabrication process of said PNC. Its working mechanism employs the PDMS matrix and CNT network which effectively and alternately cycle between contact and separation phases in the porous structure generated, by NaCl dissolution, as shown in Figure 4(b). A magnified view of the inner surface (Figure 4(c)) clearly displays the existence of exposed CNTs which are essential to the electricity-generation process.  Figure 4(d) illustrates an optical photograph of a prepared round PNC with a 5 cm diameter and 0.5 cm thickness, and with the scannning electron microscope (SEM) inset showing surface microstructure. Stomping and bending this device generated a current via various motions linked to the varying magnitude and direction of the external forces applied, meaning that it could be effectively employed to harness different motion states when worn on the insole, suggesting strong future application potential, as shown in Figure 4(e).

Overall, it ought to be remembered that a TENG that is placed directly on insoles is subjected to extremely unfavorable working conditions, including exposure to sweat and biological contaminants (bacteria) from the feet. Consequently, TENGs placed on insoles should be waterproof, possess antibacterial qualities, and be soft enough to be comfortable when placed in direct contact with the foot. In addition, considering that TENG is subject not only to vertical pressure but also to irregular lateral tearing of feet in the shoes, the TENG must be structurally sound, and the materials selected must be mechanically robust and stable.

### 3.2. TENGs Embedded in the Insoles

**Figure 5:**
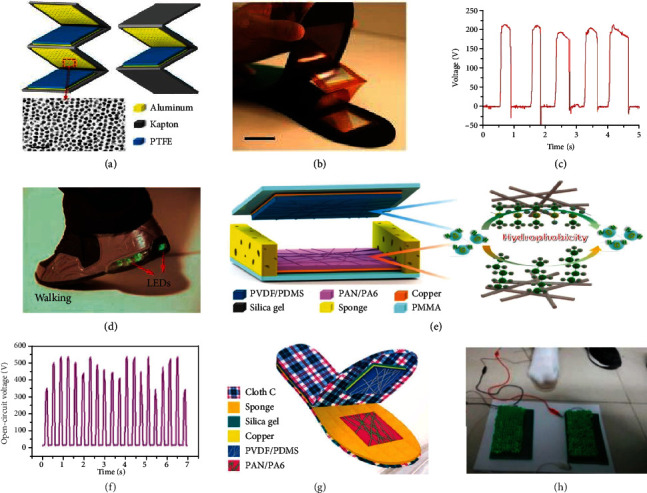
TENGs embedded in the insoles. (a) The structure of a multilayered TENG with two different viewing angles and SEM image of nanopores created on the Al foil surface. The scale bar is 200 nm. (b) Photograph of the inner structure of the insole, showing another TENG enclosed at the rear section of the insole. The scale bar is 2 cm. (c) Open-circuit voltage of the TENG. (d) Photograph of the self-lighting shoe during normal walking, showing lighted LED bulbs in the air cushion. Reproduced with permission from [163]. Copyright Elsevier, 2013. (e) Structure design of the NM-TENG. (f) Open-circuit voltage of the NM-TENG driven by human hand tapping. (g) Optical image of NM-TENG embedded in the insole. (h) The NM-TENG based power generating insole could efficiently harvest energy from human walking and light up about 400 LEDs. Reproduced with permission from [130]. Copyright Elsevier, 2017.

Some studies have reported the use of TENGs embedded into the insole, avoiding direct contact with the feet, providing protection against moisture and dirt, and significantly improving the devices' stability and robustness [51, 130, 161–163]. Zhu et al. demonstrated a self-lighting shoe, which was powered by the insole inside [163]. The TENGs were embedded in the insole to scavenge biomechanical energy during walking. As shown in Figure 5(a), the flexible multilayered TENGs were made up of three layers of TENG unit fabricated on the surface of a zigzag-shaped substrate, and the TENG unit itself was composed of a PTFE thin film and a polished aluminum (Al) foil with copper as the electrode. To increase the contact area with the PTFE film, dense nanopores were created on the Al foil surface through wet chemical etching. The energy created from the three TENG units could be coupled by parallel wiring to further enhance the output performance. Figure 5(b) shows the multilayered TENGs embedded at the heel of the insole. The same multilayered TENGs were also present in the fore-insole. The open-circuit voltage of the multilayered TENGs is displayed in Figure 5(c), where the maximum value attained reached over 220 V. The multilayered TENGs were able to light up all the LEDs installed in the sneaker during normal ambulation, as illustrated in Figure 5(d). This application not only holds the potential to provide localized lighting for outdoor activities at night, improving, for instance, personal safety, but also opens up new opportunities for harvesting pedestrian biomechanical energy.

Using a contact-separation mode, Huang et al. first demonstrated an all-fiber TENG-based insole yielding a maximum output voltage up to 240 V [162]. Considering that the surface of TENGs embedded into the insoles are easily contaminated and can be grounds for bacteria to breed, an antibacterial composite film-based TENG was also developed by researchers [161]. Employing the same working mechanism, Li et al. demonstrated a flexible and lightweight triboelectric nanogenerator (NM-TENG) constructed with a tailored nanofibrous membrane that enhanced the output performance and robustness of the device [130]. As shown in Figure 5(e), polymethyl methacrylate (PMMA) was chosen as the supporting substrates, while the nanofibrous membrane constructed TENG was composed of a layer of PVDF/PDMS nanofibrous composite membrane and a layer of PAN/PA6 nanofibrous composite membrane, using copper electrodes. The elastic sponges used in this case were added to restore the TENG shape after heel detachment and foot lifting, whereas the silica gel was laminated on the inner side of both PMMA substrates to ease mechanical buffering and adjust the gap between the triboelectric materials. As shown in Figure 5(f), the open-circuit voltage of the NM-TENG could reach a voltage as high as 540 V. Figure 5(g) illustrates the NM-TENG embedded into the insole with the ability to efficiently harvest energy from walking and light up roughly 400 LEDs, as shown in Figure 5(h). The NM-TENG provides a new and efficient pathway for designing self-powered systems, due to its cost-effectiveness, breathability, and environmental friendly material use.

**Figure 6:**
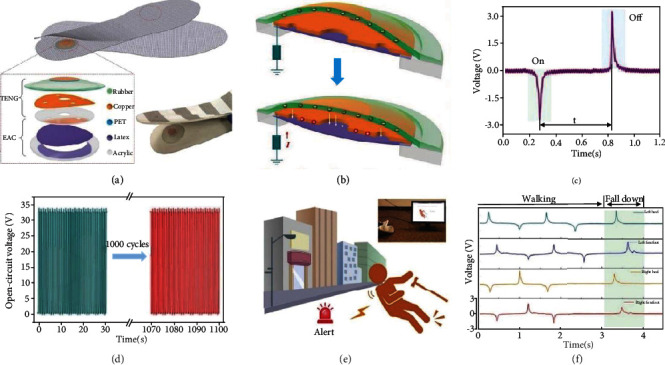
Air-pressure-driven TENG embedded in the insole. (a) The layer-by-layer structural design of the TENG and a photograph of the as-fabricated smart insole. (b) 3D illustration of the working mechanism of the TENG. (c) Signals reflecting the release (off) of foot pressure (on), on the sole. (d) The mechanical durability of the TENG after continuously working about 1000 cycles. (e) Demonstrations of the smart insoles for warning of fall down. (f) A smart shoe recording normal walking monitoring and a sudden fall. Reproduced with permission from [51]. Copyright Wiley-VCH, 2018.

Beyond strict energy production application, Lin et al. proposed an elastic TENG-based sensor embedded into the insoles possessing air-pressure-driven structural design to enable real-time gait monitoring, with a remarkably simple fabrication protocol, high durability, fast response sensitivity, and excellent mechanical robustness [51]. The TENG-based sensor consists of a TENG on the top and an elastic air chamber (EAC) made from elastic latex film at the bottom, as shown in Figure 6(a). The TENG was composed of a rubber layer as one of the triboelectric layers with a layer of copper inside to prevent interference from the environment, and a copper layer attached on top of the supporting acrylic layer to act as the other triboelectric layer. Figure 6(b) illustrates the working principle of the sensor. When the sensor is compressed by external forces, the two triboelectric layers come into contact and the internal air is squeezed into the elastic air chamber underneath. When the external forces disappear, the elastic air chamber pushes the air back into the TENG above, allowing the two triboelectric layers to achieve cyclic contact and separation during ambulation; air pressure is thus used as a suspension system. Figure 6(c) shows the voltage signal generated by one stepping cycle. Experimental studies showed that after exerting/releasing a force of 30 N for 1000 cycles, the open-circuit voltage amplitude was almost constant, suggesting excellent stability and durability, as shown in Figure 6(d). Most importantly, this sensorial approach was shown to be effectively used in a medical health monitoring setting. When an elderly person falls, an alarm can be sent out in real-time (Figure 6(e)). Figure 6(f) shows an example of a smart shoe recording normal walking monitoring and a sudden fall.

In conclusion, TENG embedded in the insole is subjected to intermittent vertical pressure during normal walking or exercise. By not coming into direct contact with the feet, embedding TENG in the insole greatly reduces the possibility of being affected by sweat and bacteria from feet. This approach presents a tidier solution than directly placing TENG onto the insole. Overall, considering that different areas of the sole are subject to different pressures profiles, passive energy harnessing can be optimized if correct engineering and placement are used.

### 3.3. TENGs Engineered into the Insoles

**Figure 7:**
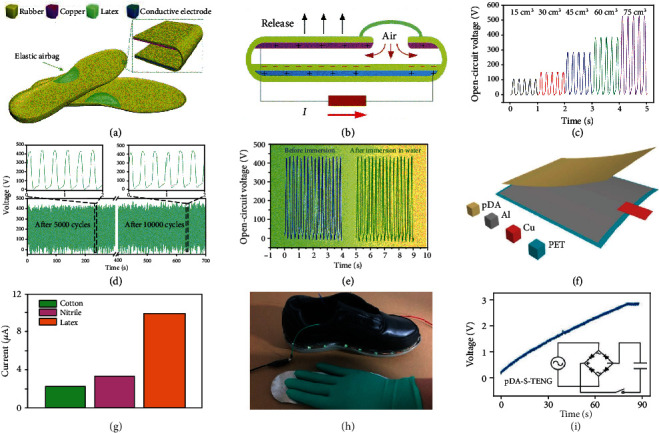
TENGs engineered into the insoles. (a) Schematic structure of the EHI. Inset: enlarged view of an inner structure of the designed insole. (b) Working principle of the energy harvesting insole (EHI). (c) Open-circuit voltage under various air volumes. (d) The durability and stability test results of the EHI after more than 10 000 cycles under the same working conditions. (e) Output voltage of the EHI before immersion and after immersion in water. Reproduced with permission from [131]. Copyright Royal Society of Chemistry, 2019. (f) Schematic illustration of the pDA-S-TENG. (g) Output currents of the pDA-S-TENG when pressed by textile, nitrile film, and latex. (h) The shiny shoe driven by the pDA-S-TENG with insole size. (i) Accumulated voltage across a single capacitor charged by this pDA-S-TENG. Reproduced with permission from [166]. Copyright Elsevier, 2018.

TENG can also be engineered directly into the insoles, but this presents considerable constraints in material selection and structure design, not only to ensure the comfort of the user but also to ensure the prolonged durability of the TENG device itself [131, 164–169]. Lin et al. developed a waterproof TENG-based smart insole to extract biomechanical energy for sustainably powering wearable electronics [131]. As shown in Figure 7(a), the TENG-based energy harvesting insole (EHI) was composed of a sealed airtight-cavity-structural TENG and an elastic crescent-shaped latex-made airbag mounted in the middle of the TENG-based insole itself. Rubber was selected as the hollow sealing material of the TENG-based insole, and copper was mounted on the inside top surface of the rubber acting as one triboelectric layer and electrode. The inside bottom surface of the rubber was the other triboelectric layer, which was coated with an electrode layer made from a mixture of silicone rubber and graphite. The working principle is displayed in Figure 7(b), and comprised an airbag connected to the airtight cavity used to drive air to the airtight cavity when external force was released, achieving cyclic contact and separation between two triboelectric materials while walking. Figure 7(c) shows that the larger the volume of the airtight cavity, the greater the open-circuit voltage became. This device presented excellent stability and durability as clearly seen in Figure 7(d). The most remarkable advantage of this approach was its waterproof capabilities, with no significant reduction in output performance after washing (Figure 7(e)).

With regards to employing multilayer structures, Hou et al. first developed a cost-effective and simple-to-fabricate TENG employing contact-separation mode for effectively harvesting ambulatory energy [169]. Instead of using a sponge as a spacer above, a liquid-metal-elastomer foam was selected as both one triboelectric material and spacer, to improve output performance [164]. Ma et al. demonstrated a polydopamine- (pDA-) modified TENG (pDA-S-TENG) that is a simple, versatile, antibacterial, and antifouling device [166]. The structure of the pDA-S-TENG is shown in Figure 7(f). This specific example consisted of a pDA membrane on the top as a triboelectric layer, an Al film in the middle as the electrode layer, and a polyethylene terephthalate (PET) film at the bottom as a substrate layer. The working method used was the single-electrode mode, which permits contact with a range of different materials to produce electricity, as shown in Figure 7(g). Latex was shown to yield the best output performance. Figure 7(h) illustrates the shiny shoe, lit by alternately patting the pDA-S-TENG based insole by hand instead of walking. The voltage of the capacitor reached about 3 V once powered by this TENG at a 5 Hz frequency, as shown in Figure 7(i). Due to its bactericidal and antifouling properties, this device promises good development potential in the field of self-powered wearable electronics. Another approach to address these latter issues includes using a simple membrane structure working in the single-electrode mode which exhibits self-sterilizing properties [168].

**Figure 8:**
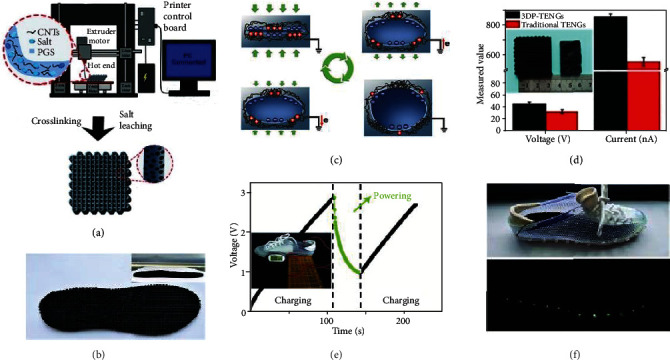
3D-Printing TENGs made into the insole. (a) Schematic diagram of the fabrication of the 3DP-TENGs and the hierarchical porous structure. (b) The images of top view and side view (insets) of a 3DP-TENG insole. (c) Working principles of a single pore. (d) The comparison of output performance of the 3DP-TENGs and the traditional TNEGs. (e) Voltage feature of a 3DP-TENG insole charged 22 *μ*F capacitor which is powering the electronic watch. Image of the self-charging system to power electronic watch (inset). (f) Photograph of a 3DP-TENG insole simultaneously lighting LEDs of a self-powered lighting shoe inside (top). Reproduced with permission from [167]. Copyright Wiley-VCH, 2018.

The most creative integration structure within a regular shoe, is that of Chen et al., who developed a 3D-printed TENG (3DP-TENG) with a simple integrated procedure, which can be easily, widely and effectively used in making smart insoles [167]. The fabrication process of the 3DP-TENG is shown in Figure 8(a), and the top view as well as a side view (insets) of a 3DP-TENG insole is displayed in Figure 8(b). The device was composed of poly (glycerol sebacate) (PGS) as one triboelectric material and CNTs as the other triboelectric material and electrode, working in the single-electrode, mode as shown in Figure 8(c). Salt particles were added to the PGS to obtain a hierarchical porous structure after salt leaching. Compared with traditional molding methods, the hierarchical porous 3DP-TENGs could achieve better output performance using the same amount of composite ink, as shown in Figure 8(d). To prove its practicality, experiments were run as shown in Figure 8(e), which shows the voltage property of a 22 *μ*F capacitor being charged by the 3DP-TENG insole simultaneously powering an electronic watch. A self-powered lighting shoe shown in Figure 8(f) shows that the LEDs can be lit by stomping through the 3DP-TENG insole inside the shoe. The 3D printing strategy developed here has broad development prospects, instead of assembling different parts together to obtain 3D structures like the previously reported TENGs.

In summary, the TENG used directly as an insole is relatively large in size and its activity varies from place to place in relation to applied foot pressure. With the entire insole size area being in contact with the soles of the feet, this form is most affected by the sweat, dirt, and bacteria produced by the soles of the feet. Therefore, the requirements for material selection of this form of TENG must be of the highest quality, water-resistant, bacteria-resistant, structurally stable, and mechanically robust. Future research could also integrate human sweat and temperature sensors with TENG to power them and send wireless signals to mobile phones to provide information for human health assessments.

### 3.4. TENGs Integrated into the Soles

**Figure 9:**
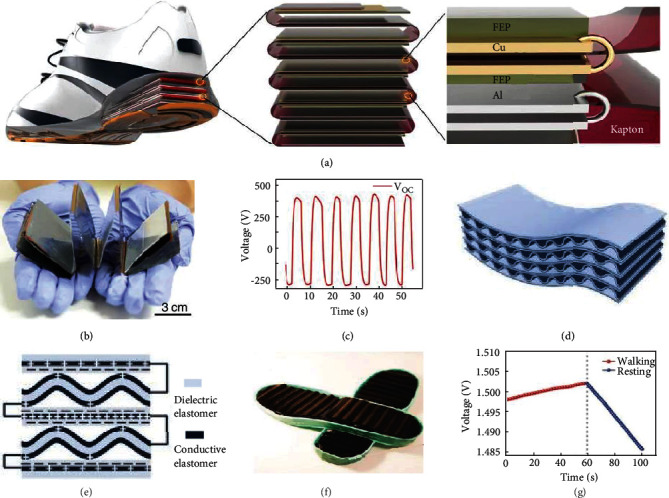
TENGs integrated into the soles. (a) Structure of the designed multilayer TENG. (b) Photo of an as-fabricated TENG. (c) Open-circuit voltage output of the as-fabricated TENG. Reproduced with permission from [132]. Copyright Springer Nature, 2015. The 3D schematic illustration to demonstrate the structure of the TENG. (e) Working principles of the multilayer elastomeric TENG. (f) The photo showing the multilayer elastomeric TENG integrated into the sole. (g) The photo to demonstrate the function of the fitness tracker that is powered by the TENG-based self-charging power system. Reproduced with permission from [171]. Copyright Wiley-VCH, 2017.

TENG can also be integrated into the sole during the production process, given that the sole has a relatively large space that can harness large movement ranges and improve output performance [16, 132, 170, 171]. Niu et al. first proposed a highly-efficient self-charging system for sustainably powering wearable electronics, in which the most important part was the ingenious design of TENG [132]. As shown in Figure 9(a), a zigzag-shaped Kapton film was selected as the substrate and decorated with multiple TENG units, which were composed of Al foil as both the triboelectric layer and electrode and fluorinated ethylene propylene (FEP) layer with the copper electrode. The as-fabricated TENG is very thin and lightweight (Figure 9(b)), thus easily embedded into the soles. Figure 9(c) shows that the output voltage was able to reach up to 700 V, indicating great application potential. A novel corrosion-resistant copper-nickel based TENG with a similar, multilayered, and stacked structure that can deliver up to 1500 V when integrated into the shoe, was also developed [170].

Another developed TENG structure also relied on the multilayer approach with closely stacked arches to improve output performance [171]. Figure 9(d) illustrates the structure of the multilayer TENG made up of many planes and waved layers. Every plane layer is the structure of dielectric–conductive–dielectric style, and every waved layer is the structure of conductive–dielectric–conductive architecture, as shown in Figure 9(e), to provide a nifty TENG design. The Ecoflex 00-30 super soft silicone was selected as the dielectric elastomer, and a mixture of Ecoflex 00-30 with carbon black and carbon nanotubes was selected as the conductive elastomer. The working mechanism developed relied on cyclic changes of contact between the dielectric elastomer and conductive elastomer, driven by an external pressure or a stretchable force. Figure 9(f) displays TENG integrated into the sole, which can continually power a pedometer while walking at a normal frequency (Figure 9(g)). The TENG not only has high efficiency in generating electricity but can also be combined with a pedometer and fitness tracker to monitor human movement data, presenting very broad practical application value.

In conclusion, the TENG is integrated into the sole and can be completely isolated from the shoe interior and the external environment, resulting in extremely superior working conditions. In order to allow comfortable wear, the sole is generally designed to be thicker and elastic. This makes it possible for multiple TENGs to work together in contact-separation mode, which not only effectively utilizes the elasticity of the sole for intermittent contact and separation, but utilizes it also to work with multiple TENGs simultaneously and improve output efficiency. In addition, future research suggests designing the entire sole as a TENG to increase the area of contact and thus the output power. Due to the excellent working conditions inside the soles, it is also possible to integrate locator chips into the soles and use TENG for permanent power supply, particularly suitable for real-time location monitoring of the elderly and children.

### 3.5. TENGs under the Soles

**Figure 10:**
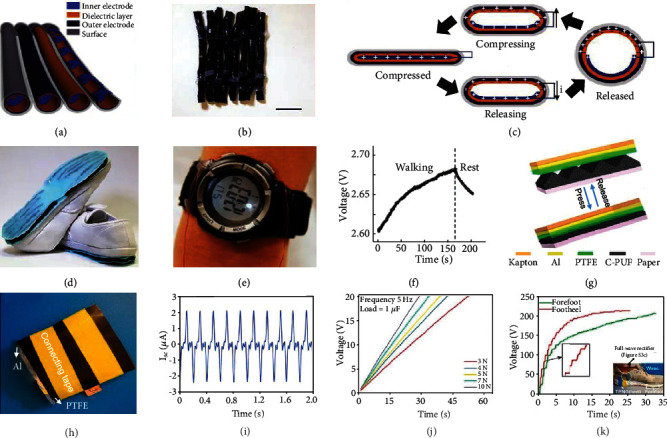
TENGs under the soles. (a) Structure sketch of the TENG tubes. (b) Photograph showing TENG tubes in diameter of 2–3 mm weaved into textile. (c) Working mechanism of the TENG. (d) Image of the “energy-shoe.” (e) An electronic watch is driven by the textile TENG. (f) A LIB is charged simultaneously by the “energy-shoe” while walking. Reproduced with permission from [133]. Copyright Springer Nature, 2016. (g) Simplified structure and working mechanism of the TENG. (h) Top view of an as-fabricated TENG. (i) Short-circuit current in short circuit mode of the TENG. (j) Voltage of the storage capacitor (1 *μ*F) versus time under different press forces with a constant frequency of 5 Hz. (k) Output voltage of the capacitor with full-wave rectifier. Reproduced with permission from [175]. Copyright Elsevier, 2018.

Some researchers have placed TENG under the soles of shoes, where its durability is greatly reduced due to severe wear and tear on the ground, and where it was thus necessary to select triboelectric or packaging materials with good mechanical robustness [133, 172–176]. In this space, most of the research achievements have been linked to generating electricity. Wang et al. developed a TENG with outstanding structural design and optimized materials [133].  Figure 10(a) shows the structure of a tube-shaped TENG, made up of a tube-shaped dielectric layer with a back electrode outside and a belt-like helix inside acting as a triboelectric layer and electrode. The silicone rubber was selected as the encapsulation material to provide flexibility and stretchability in multidimension. Silicone rubber, carbon black and CNTs were mixed to fabricate the inner and output electrodes.  Figure 10(b) shows the TENG-tubes with a diameter of 2–3 mm, weaved into textile. The working principle of the TENG-tube relies on the alternating contact/separation between the inner and outer dielectric, when compressed and released, as illustrated in Figure 10(c). To demonstrate its practical value, 40 tubes were mounted under the shoes (Figure 10(d)).  Figure 10(e) illustrates an electronic watch that can be immediately and sustainably powered by walking and a LIB which can be also charged simultaneously while walking (Figure 10(f)). Zhang et al. proposed an advanced contact-separation mode TENG, based on macroshaped and commercial conductive polyurethane foam [175]. As shown in Figure 10(g), the TENG was composed of a macrostructured conductive PU foam (C-PUF) doped with conductive carbon black powder and shaped with 5 triangle prisms acting as one triboelectric layer and electrode. Moreover a PTFE film stuck onto the top Al electrode acted as the other triboelectric layer. The Al electrode and the C-PUF were pasted to an insulating Kapton substrate and a paper substrate, respectively.  Figure 10(h) shows a top view of the as-fabricated TENG. The working mechanism here relied on the cyclic contact and separation between the PTFE and the C-PUF. The maximum short-circuit current was about 2.2 *μ*A, as exhibited in Figure 10(i). Moreover, it was discovered that the greater was the external force applied, the greater the output voltage became, as shown in Figure 10(j). When the TENG was placed under the sole to demonstrate its performance, the output voltage of the capacitor with a full-wave rectifier (as shown in Figure 10(k)) generated a greater output than at the forefoot, due to greater pressure discharge and reduced cushioning. Moreover, TENGs with a liquid PEDOT:PSS electrode [172] and with humidity-resisting characteristics [176] were also designed to simply generate electricity.

**Figure 11:**
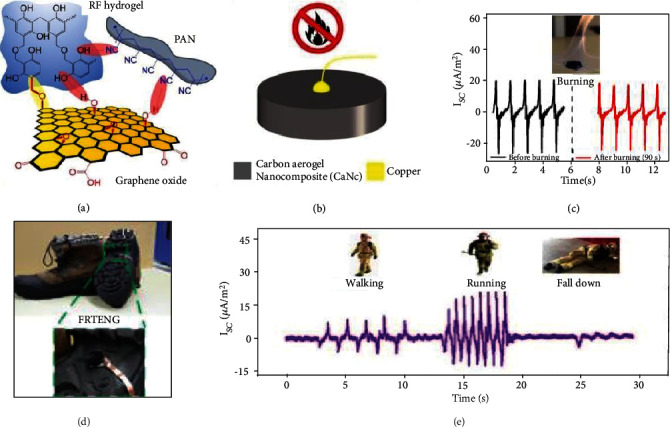
Fire-retardant and self-extinguishing TENG under the sole. (a) The fabrication process of a hybrid carbon aerogel with functionalized nanofibers/nanosheets. (b) Schematic diagram of a single-electrode mode of FRTENG. (c) Short circuit current density comparison before and after burning. Inset: shows the flammability test of the FRTENG in 90 s. (d) The image showing FRTENG integrated onto the firefighter's sole. (e) Temporal changes of the vector sum during the various movements of the firefighter. Reproduced with permission from [174]. Copyright Elsevier, 2019.

TENGs integrated under the soles of shoes have other surprising effects. For instance, Ahmed et al. developed a fire-retardant TENG (FRTENG), which could endure extremely high temperatures [174]. The FRTENG consists solely of a copper wire and carbon aerogel nanocomposite (CaNC) working in the single-electrode mode. This mechanism was based on sol-gel polymerization of resorcinol-formaldehyde, mixed with polyacrylonitrile nanofibers and graphene oxide nanosheets (Figure 11(a)). After the sol-gel was carbonized in a supercritical drying step, lightweight and durable carbon aerogel was obtained (Figure 11(b)). This could be directly fabricated into FRTENG, working in the single-electrode mode. Figure 11(c) illustrates how the FRTENG is flame resistant, and how the value of short-circuit current is only slightly reduced after fire exposure. In addition, the FRTENG could be mounted under the shoes (Figure 11(d)) as a tracking sensor to wirelessly monitor firefighters under dangerous and hazardous conditions, allowing to automatically signal for help in an emergency. The short circuit current obtained is shown in Figure 11(e); the variation in both amplitude and frequency could indeed be used to distinguish the walking, running, and falling of a fireman.

In general, TENG is exposed to extremely harsh working conditions when placed under the sole of a shoe, and its service life is greatly reduced by the crushing pressure of the sole of the foot, as well as the severe friction of the pavement. Therefore, future research should focus on the encapsulation of TENG. The packaging material ought to have strong mechanical robustness, wear resistance, and water resistance. In addition, the structural design of the TENG ought to be optimized to collect both the downward pressure of the foot and the friction between the shoes and the ground, thus significantly increasing the power output that can be used for night lighting and the continuous power supply of other electronic devices.

On the basis of the above discussion, TENG based smart shoes could be designed and engineered in a manner that allows installing them into commercial shoes without negatively affecting wearing comfort. The electricity generated from the smart shoes can, not only provide continuous power to various wearable bioelectronics, but also be employed as a self-powered sensors to monitor our walking gait in real-time. The output performance and main features of various TENG enabled smart shoes are summarized in Table 1.

## 4. Summary and Perspective

**Figure 12:**
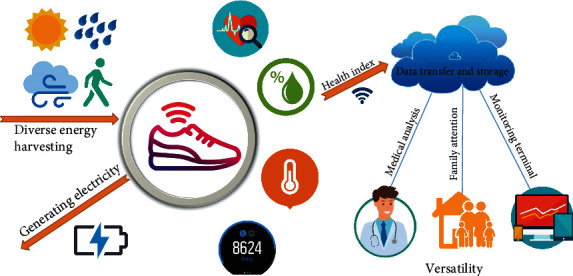
The direction of the future development of TENG enabled smart shoes. Diverse energy harvesting: in addition to harvesting energy from walking, we can envision harvesting energy from light, wind, and rain. Intelligence: a real-time collection of human health information, including heart rate, humidity, temperature, and step count, and pressure. Versatility: in addition to collecting human health data, the TENG device can also send data to the doctors for continuous health monitoring.

Evergrowing device interconnectability, as well as the pervasive emergence of next generation Internet of Thing infrastructure, provides an ecosystem in which wearable electronics will flourish, changing our lives in ways we are yet to understand. In order to convert passive human biomechanical energy into electricity, triboelectric nanogenerators can be employed to provide a sustainable and pervasive energy solution, and they can be harnessed to help materialize the aforementioend Internet of Things paradigm. Among them, research into TENG-based smart shoes has attracted significant attention due to the ability to harvest the highest amount of available passive biomechanical energy released during locomotion. In this review, the latest achievements of TENG-based smart shoes for biomechanical energy harvesting are systematically summarized and reviewed from two perspectives (Figure 12). The foremost is that smart electricity generation shoes are a sustainable and pervasive power source for wearable electronics, a secondly that. The other is that they can also monitor human health status by analyzing the generated electric signals.

Although research into TENG-based smart shoes has achieved remarkable progress, as an emerging energy technology with great potential, both challenges and opportunities coexist. To advance the field development, research efforts could be focused on improving the following aspects:

1. Wearing Comfort. The comfort of the insole largely determines whether it could be acceptable for daily wearing. Therefore, in the design of TENG, especially in the interior of shoes, new materials that are soft, breathable, and mechanically durable are highly desired. In addition, new soft electrodes need to be developed to replace the rigid metal electrode to improve wearability.
2. Enhancing the Waterproof Capabilities. Human feet perspire heavily, resulting in a relatively high internal humidity of the shoes, which impacts TENG output negatively [177]. Therefore, it is particularly necessary to develop TENG that are waterproof or which are optimized in functionality in humid environments. Enhancing the breathability of the shoes could be another approach to reduce the perspiration induced internal humidity. In addition, the waterproof property of smart shoes is also necessary against pluvial weather. TENG and other electronic devices can be significantly affected by water leaking into shoes, so the easiest way to solve this problem is to apply a waterproof coating to the outside of the smart shoes to prevent rain from penetrating.
3. Enhancing the Mechanical Durability. TENG-based smart shoes can be subject to considerable mechanical stress due to repeated and constant body movement which could place a significant impact on the mechanical durability of the shoes, and especially the triboelectric materials. Thus, highly durable materials with enhanced wearability are required. Robust structure design could be another pathway to enhance the mechanical strength of the smart shoes.
4. Detachable Property of TENG. Smart shoes require constant washing. Besides enhancing the washability of the TENG component of the shoes, an alternative approach is to make the TENG sole component easily disassembled and assembled. TENG devices could be detached when the shoe is washed and later reinstalled when the shoe is dried.
5. Structure Optimization according to the Working Location of the TENG. Foot pressure varies during the various stages of ambulation, and when designing footwear-enabled TENG, the characteristics of the distribution of human foot pressure should be considered to guide the TENG structure and optimize the conversion efficiency of mechanical energy into electrical energy, improving the overall efficiency of energy collection.
6. Energy Harvesting beyond Human Footfall. Existing smart shoes can convert human biomechanical energy into electricity. However, other forms of renewable energy are also accessible in the ambient environment, including raindrop striking on the outside of the shoe, wind blowing, and even snow friction, which all can also be converted into electricity while walking.
7. Intelligence. The rapid advancement of modern technologies and artificial intelligence is changing our way of living. Intelligent and multifunctional smart shoes could be explored beyond electricity generation. For instance, in order to automatically control the temperature of an inner space, smart shoe could be employed to control the heating, cooling, or ventilation automatically, and maintain the thermal comfort for an individual. Additionally, smart shoes could also be applied to monitor human body movement and send real-time health data wirelessly to the medical system for personalized health care.

## Figures and Tables

**Table 1:** The output performance and main features of the TENG enabled smart shoes.

TENG's position	Structural design features	Main properties	Voltage	Current	Power	Duration	References
On the insole	Textiled TENG with plastic metal electrodes	Flexible and stable	30.96 V	3.07 *μ*A	13.97 *μ*W	7200	[158]
	Multilayer TENG with porous nanocomposite	Stretchable	55 V	170 nA	—	—	[159]

Embedded into the insole	Multilayer TENG with a zigzag-shaped substrate	Flexible	220 V	600 *μ*A	—	—	[163]
	Simple TENG with nanofibrous membrane	Breathable, lightweight and flexible	540 V	110 *μ*A	—	6000	[130]

Engineered into the insole	An airtight-cavity-airbag structural insole	Waterproof and durable	528 V	81.2 *μ*A	5.47 mW	10000	[131]
	pDA-modified TENG	Simple, antibacterial, and antifouling	80 V	28.8 *μ*A	311.3 *μ*W	—	[166]

Integrated into the sole	Multilayer TENG with a zigzag-shaped substrate	High-efficient and lightweight	700 V	—	1.044 mW	—	[132]
	Multilayer elastomeric TENG with closely stacked arches	High mechanical robustness and waterproof	—	16.2 *μ*A	—	200000	[171]

Under the sole	Tube-shaped TENG	Waterproof and anticorrosive	140 V	—	—	—	[133]
	TENG based PU foam and PTFE	Soft and lightweight	120 V	2 *μ*A	—	—	[175]
